# Macrophages on the Peritoneum are involved in Gastric Cancer Peritoneal Metastasis

**DOI:** 10.7150/jca.31787

**Published:** 2019-08-29

**Authors:** Hongjiang Song, Tie Wang, Lining Tian, Shuping Bai, Li Chen, Yanjiao Zuo, Yingwei Xue

**Affiliations:** 1 Department of Gastrointestinal Surgery, Harbin Medical University Cancer Hospital, Harbin, Heilongjiang Province, China; 2 Department of Medical Education, First Affiliated Hospital of Harbin Medical University, Harbin, Heilongjiang Province, China; 3 Department of Internal Medical Oncology, Harbin Medical University Cancer Hospital, Harbin, Heilongjiang Province, China

**Keywords:** gastric cancer, peritoneal metastasis, macrophages, tumor microenvironment

## Abstract

Tumor-associated macrophages (TAM) have been shown to support tumor growth and progression by various mechanisms. However, the roles of TAM in gastric cancer (GC) peritoneal metastasis remain elusive. To explore the roles of macrophages in the process of GC peritoneal metastasis, we performed the present study. Samples from the primary GC tumor beds, surgical margins, peritoneal metastatic lesions and surrounding tissue, and the Pouch of Douglas, were collected, fixed by formalin, and embedded with paraffin. Immunohistochemistry staining for macrophages markers was performed. The peritoneal lavage was obtained from a fraction of patients to analyze the ratios of epidermal growth factor (EGF)- and vascular endothelial growth factor (VEGF)-secreting macrophages in the peritoneal cavity. GC patients with peritoneal metastasis had increased levels of macrophages and alternatively activated macrophages in the peritoneum compared to those without dissemination. Patients bearing more macrophages in the peritoneum had a poorer prognosis. GC patients bearing peritoneal metastasis harbored an increased level of angiogenesis. Macrophages in the peritoneal cavity were a source of EGF and VEGF.

Macrophages in the peritoneum of GC patients play a supportive role for peritoneal metastasis by producing EGF and VEGF. Macrophages in the peritoneum might be a therapeutic target in the future.

## Introduction

Gastric cancer (GC), one of the most common cancers worldwide, marks the 4^th^ most common rank of prevalence and the 2^nd^ highest rate of cancer-related mortality 1-4. The prognosis of GC patients is poor, whose 5-year survival rate is less than 20%, despite radical surgery can be applied to those with the early stages of the disease 5-7. Even received an R0 (no residue tumor) radical resection, GC patients still have a relatively high risk of recurrence. A retrospective analysis for GC patients in the United States showed that 42% of GC patients undergoing an R0 resection had at least one recurrent event after surgery, among whom 76% were detected within 2 years after surgery 8. In patients with recurrent GC, 29% have peritoneal metastasis, which represents the most prevalent pattern of relapse 9.

Peritoneal metastasis of GC is one of the incurable factors 10. A fraction of GC patients are diagnosed with peritoneal dissemination before or during the operation, despite an increased number of early GC patients are being detected by a routine screening program 7. A Japanese cohort 9 demonstrated that the peritoneum was the most frequent site of post-operative recurrence. The outcome of GC peritoneal metastasis patients is extremely poor, whose median overall survival was reported to be only 3.1 months if left untreated 11. Although strategies for GC peritoneal spread have been developed, such as peritoneal cytoreductive surgery plus local or systemic chemotherapy 12, the outcome remains pessimistic. More importantly, molecular mechanisms of GC peritoneal metastasis are poorly understood.

As another half of cancer, cancer stromal cells play an important role during tumor initiation and progression 13, 14. Cancer-related inflammation is an imperative component of the cancer stroma 13, 15-17, where tumor-associated macrophages (TAM) are critical for supporting tumor growth and metastasis 18, 19. Published clinical data have demonstrated that the abundance of TAM is negatively related to the prognosis in prostate, ovarian and cervical cancer patients, but the data for GC are contradictory 20. TAM support tumor growth and progression by releasing growth factors, proteolytic enzymes, cytokines, chemokines, and other mediators, which are critical for angiogenesis and tumor metastasis 19, 21. However, the above-mentioned mechanisms of TAM have not been tested in the context of GC peritoneal metastasis.

Although TAM have been shown to be supportive for tumor aggression during GC angiogenesis 22, the relationship of TAM and GC peritoneal metastasis remains to be unappreciated. Therefore, the current study aimed to test the hypothesis that TAM could have supportive effects during the process of GC peritoneal metastasis. We demonstrate here that macrophages in the peritoneum rather than the primary tumor site play a critical role in supporting metastatic tumor cells to develop by producing EGF and VEGF.

## Materials and Methods

### Patients

Between October 2010 and August 2013, patients who were diagnosed with GC and received surgery as the initial treatment in our department were enrolled in this study. All patients obtained the histological confirmation of gastric adenocarcinoma and computerized tomography (CT) scan assessments of negative remote metastasis before surgery. The criteria for recruitment of patients with peritoneal metastasis was that a clear peritoneal lesion could be removed for histological diagnosis, which was subsequently verified to be metastatic cancer; whereas the criteria for non-peritoneal metastasis were: (1) no obvious peritoneal spread was observed during surgery; (2) no ascites, and the peritoneal lavage led to a negative cytological result; (3) frozen sections for suspect lesions of the peritoneum showed no evidence of metastasis; and (4) patients had more than 2 years of cancer-free survival after surgery. In order to make recruited patients comparable between groups, only patients with the subserosa invasion (T3) were involved in this cohort. The disease stages of gastric cancer in this cohort were classified according to the Japanese Classification of Gastric Carcinoma--3rd English Edition 23. The characteristics of patients have been summarized in Table 1. All patients were followed up until death or up to 5 years after surgery.

### Ethical Considerations

The Ethics Committee of Harbin Medical University Cancer Hospital approved and supervised the research proposal (approval number: 20100076). Written informed consent forms were explained, agreed, signed, and obtained from each individual.

### Histological Specimens

Surgically resected samples, including the primary tumors, surgical margins, suspicious peritoneal metastatic lesions, normal peritoneal tissue adjacent to the corresponding possible peritoneal metastasis, and a biopsy from the pouch of Douglas, were collected immediately after removal, fixed with formalin and subsequently embedded with paraffin. All specimens were cut with 4- to 6-μm of thickness and stained with hematoxylin and eosin. The histological diagnosis and grade were performed according to the Japanese Classification of Gastric Carcinoma-- 3rd English Edition 23.

### Immunohistochemistry

Consecutive 4- to 6-μm sections were cut from each specimen and stained with the corresponding primary antibodies. The primary antibodies used in this study included CD68 (a marker for macrophages), CD163 (a marker for M2 macrophages), CD34 (a marker for progenitor endothelial cells as well as neo-angiogenesis 24), epidermal growth factor (EGF), and vascular endothelial growth factor (VEGF). Information regarding manufacturers and titration of antibodies was listed in Table 2. Corresponding isotype control antibodies were served as background staining. Slides were assessed by two pathologists blindly on a multi-headed microscope, using a similar approach published previously 25. The numbers of CD68-, CD163-, and CD34-positive cells per 400
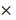
high-power field (HPF) were counted. Five random high-power fields per sample were viewed for scoring. The results by the pathologists were subsequently verified by a computer program (ImageJ, Windows Edition, National Institute of Health, Bethesda, MD, USA) to exclude human errors.

### Flow cytometry

The peritoneal lavage was obtained from a fraction of patients in our cohort (20 patients without peritoneal metastasis, and 20 with metastasis), according to a published paper 26. Biopsy samples of the pouch of Douglas were dissociated to achieve single-cell suspensions. Cells were incubated with FC blockers (eBioscience, Thermo Fisher Scientific, Waltham, MA, USA) before incubation with targeted antibodies. Macrophages were defined as CD45^+^CD68^+^ (florescence-conjugated antibodies, PE and FITC, respectively; both from eBioscience). Intracellular staining of EGF and VEGE were performed by using the corresponding fluorescence APC-conjugated antibodies (eBioscience) and a permeabilization-fixation kit (eBioscience). The titrations of antibodies were used based on the manufacturer's instructions. A total of 10,000 cells was applied using an Original Attune Flow cytometer (Applied Biosystems, Foster City, CA, USA). The data were analyzed by Kaluza software 1.3 (Beckman Coulter, Brea, CA, USA). The proportions of CD45^+^CD68^+^EGF^+^ cells in CD45^+^ cells and CD45^+^CD68^+^VEGF^+^ cells in CD45^+^ cells were calculated and compared between patients with and without peritoneal metastasis.

### Statistical analysis

Data were analyzed using GraphPad Prism 6 (GraphPad Software, San Diego, CA, USA). The equality of comparisons between proportions was analyzed using Fisher's exact test. The numbers of CD68-, CD163- and CD34-positive cells were presented as mean±SEM (the standard error of means) and examined with the Student *t* test. The rank data was analyzed by Spearman correlation tests. Non-parametric parameters were compared by the Mann-Whitney U test. Kaplan-Meier survival curves were created and compared by the log-rank statistic. The significance level of the analysis was set to a *p* value of less than 0.05.

## Results

### The abundance of macrophages in the peritoneum rather than that in the primary tumor site was related to gastric cancer peritoneal metastasis

**Figure 1:**
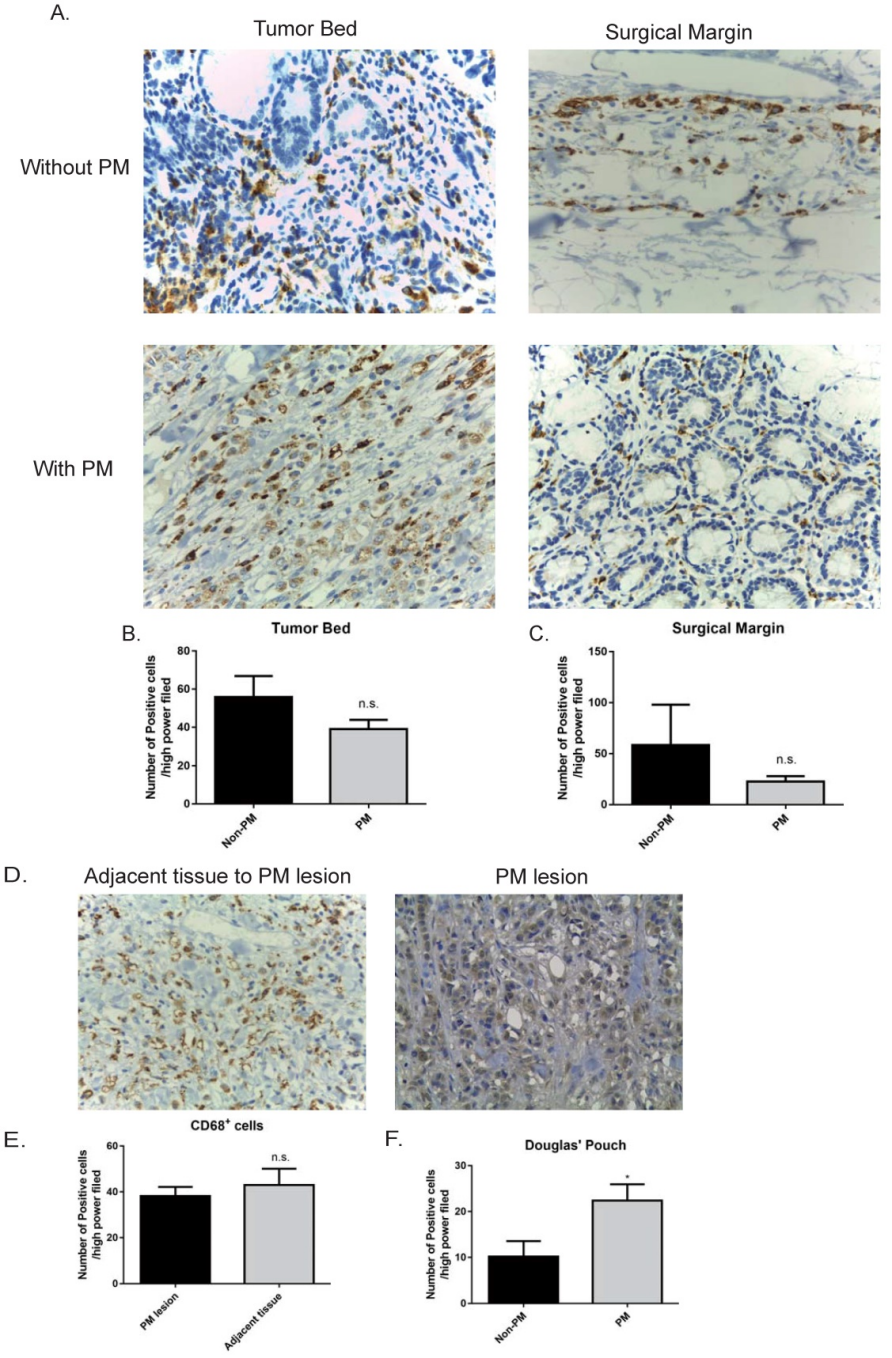
** GC patients had increased levels of CD68^+^ macrophages in the peritoneum, rather than the tumor bed and surgical margin.** A. Representative pictures of immunohistochemistry staining of CD68 (stained in brown) in the specimens (400×). The numbers of CD68^+^ cells in the primary tumor bed (B), surgical margin (C) showed no statistical significance between the GC patients without peritoneal metastasis and those with peritoneal metastasis. In GC patients with peritoneal metastasis, peritoneal metastatic lesions had a similar number of CD68^+^ cells compared to the adjacent peritoneal tissue (D and E). F. GC patients with peritoneal metastasis harbored more CD68^+^ cells in the Pouch of Douglas with respect to GC patients without peritoneal metastasis. GC: gastric cancer. PM: peritoneal metastasis. n.s., no significance; *, *p*<0.05, by student *t* tests.

The expression of CD68, a marker of macrophages 27, was measured in the primary tumor bed, surgical margin, and peritoneal metastatic lesions as well adjacent peritoneal tissue (5 cm from the margin of a lesion). We expected a higher level of CD68 expressed in the primary tumor of GC patients with peritoneal metastasis than those without spread in the peritoneum. To our surprise, the abundance of macrophages in the primary tumor bed and that on the surgical margin had no difference between GC patients with peritoneal metastasis and those without metastasis (no peritoneal metastasis v.s. peritoneal metastasis: tumor bed, 55.83±11.08 v.s. 39±4.91/HPF, *p*>0.05; surgical margin, 58.4±39.72 v.s. 22.6±5.29 /HPF, *p*>0.05. Fig. 1 A, B, and C). We also compared the abundance of CD68^+^ macrophages in the peritoneal metastatic lesions and the adjacent normal mesothelium tissue. As shown in Fig. 1 D and E, the abundance of CD68^+^ macrophages in the metastatic lesions were comparable to that of adjacent peritoneal tissue (normal tissue v.s. metastatic tumor, 38.2±3.967 v.s. 43±7.134, *p*>0.05). Last, we examined the number of macrophages in the Pouch of Douglas. A standard procedure was performed, where a small piece of tissue (3×3 mm) was resected from the centre of the bottom of the Pouch of Douglas regardless of the presence of tumors or not. Patients with peritoneal metastasis harbored increased numbers of macrophages in the Pouch of Douglas compared to those without peritoneal metastasis (no metastasis v.s. peritoneal metastasis, 10.2±3.36 v.s. 22.4±3.57 /HFP, *p*<0.05, Fig. 1 F). These data indicate that macrophages in the peritoneum (the Pouch of Douglas as an avatar) play a more important role during the process of peritoneal metastasis of GC patients with respect to macrophages in the primary tumor sites, as the only difference in the abundance of macrophages was observed in the Pouch of Douglas between patients with and without peritoneal dissemination.

### Increased numbers of CD68^+^ macrophages in the peritoneum were correlated with poorer postoperative overall survival in GC patients

**Figure 2:**
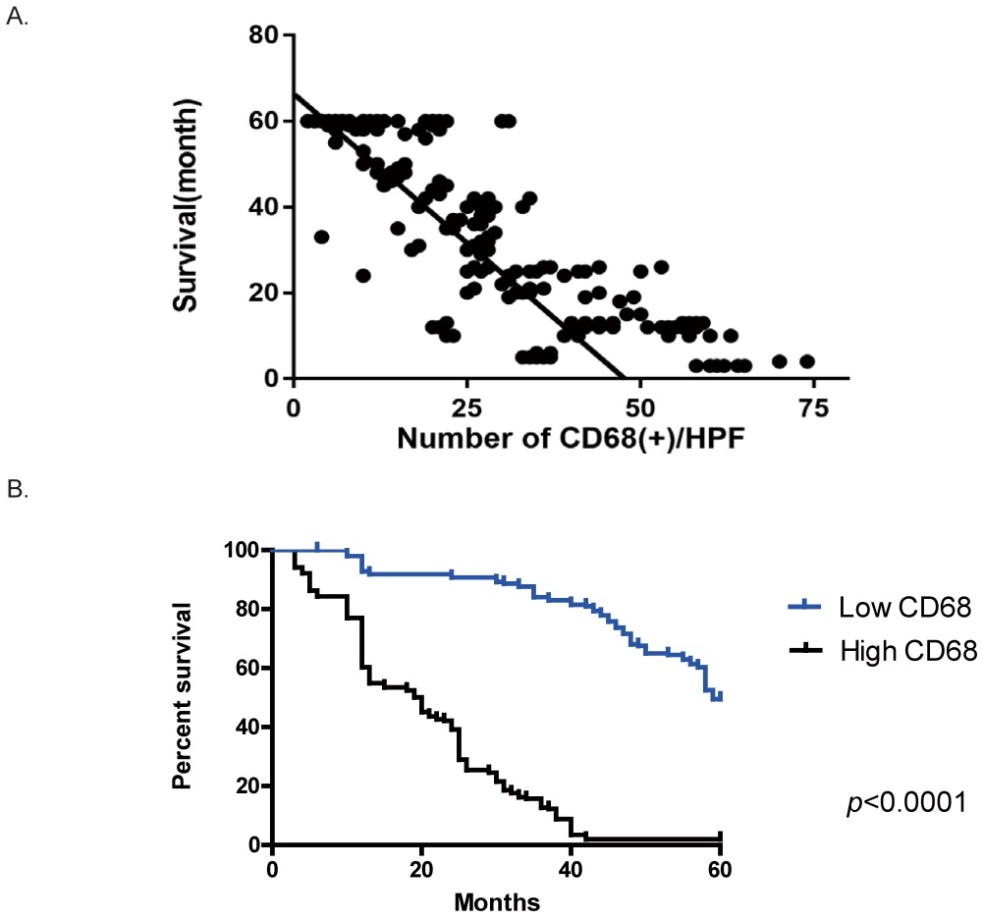
** An increased number of CD68^+^ cells in the Douglas' Pouch of GC patients was related to a poorer survival.** A. The correlation between the number of CD68+ cells per HPF and the overall survival after operation. The number of CD68^+^ cells was negatively correlated with overall survival. *p*<0.0001, by Pearson r tests. B. The survival curves of the groups of low CD68+ cells in the Douglas' Pouch (<25/ HPF) and high CD68+ cells in the Douglas' Pouch (≥25/HPF), *p*<0.0001, by Log-rank tests.

We also observed the correlation between the abundance of macrophages and the prognosis of GC patients. In our cohort, no correlation was seen between the number of macrophage in the primary sites or surgical margins and the outcome (data not shown). However, with respect to the correlation between CD68^+^ macrophages in the Pouch of Douglas and metastasis, the number of CD68^+^ cells on peritoneum was correlated with a poor prognosis. Patients harboring a higher number of CD68^+^ cells on peritoneum had a worse outcome (Figure 2 A, r= -0.84, 95% confidence interval (CI) [-0.8666, -0.8085], R^2^=0.7055, *p*<0.0001). We divided our cohort into two groups according to the median of CD68^+^ cells/HPF in the Pouch of Douglas across the entire cohort (24.95/HPF), which were the low CD68 group (<25/HPF) and the high CD68 group (≥25/HPF). As shown in Figure 2 B, the high CD68 group had a shorter postoperative overall survival compared to the low CD68 group (p=0.0112). These results suggest that the number of CD68^+^ cells on GC patients' peritoneum is a predictive parameter for the overall survival.

### The abundance of M2 macrophages in the Douglas Pouch was correlated to gastric cancer peritoneal metastasis

**Figure 3:**
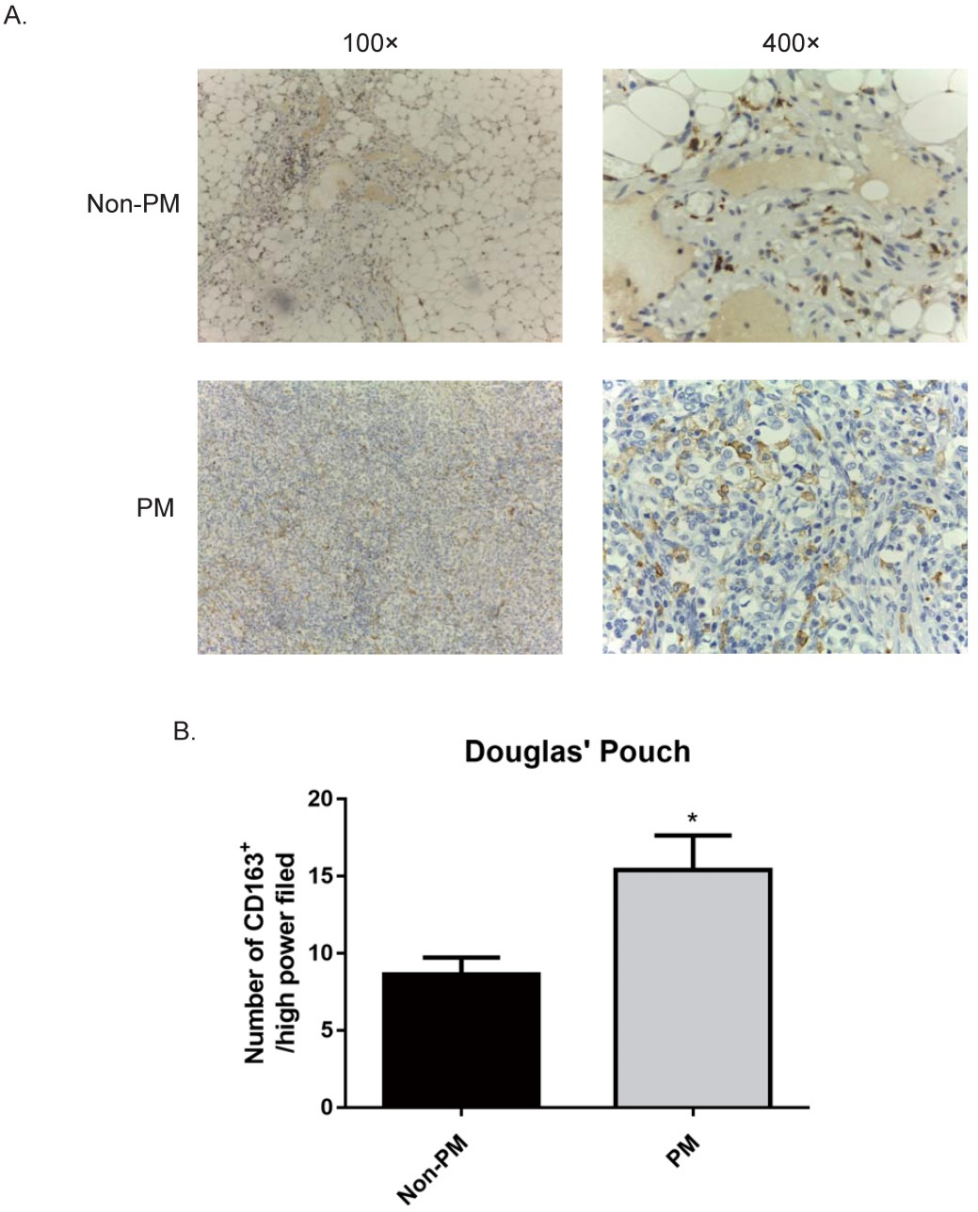
** GC patients with peritoneal metastasis had an increased number of CD163^+^ M2 macrophages in the Pouch of Douglas.** Samples of the Douglas' Pouch were collected from GC patients without and with peritoneal metastasis, and CD163^+^ M2 macrophages were detected by immunohistochemistry staining. A. Representative images of immunohistochemistry of CD163^+^ cells (stained in brown). B. Patients with peritoneal metastasis had more CD163^+^ M2 macrophages. *, *p*<0.05, by student *t* tests.

Since TAMs have an M2 macrophages phenotype, we checked the M2 marker (CD163) in the primary tumor site, surgical margin and peritoneum by IHC staining. In line with the results of CD68^+^ cells, the abundance of CD163^+^ M2 macrophages in the primary tumor site and surgical margin showed no correlation with peritoneal metastasis (data not shown). Meanwhile, the number of CD163^+^ cells in the Pouch of Douglas of GC patients had a positive association of peritoneal spread. The patients with peritoneal metastasis had an increased number of CD163^+^ cells on the peritoneum compared to the metastasis-free patients (no peritoneal metastasis v.s. peritoneal metastasis, 8.6±1.12 v.s. 15.4±2.24, p<0.05, Fig. 3). These results demonstrated that CD163^+^ M2 macrophages support the progression of GC peritoneal metastasis. However, we failed to detect a correlation between prognosis and the abundance of CD163^+^ cells in the primary tumor site, surgical margin or peritoneum, suggesting CD163 is not a predictive marker for the overall survival.

### GC patients with peritoneal metastasis had more progenitor endothelial cells in the peritoneum

**Figure 4:**
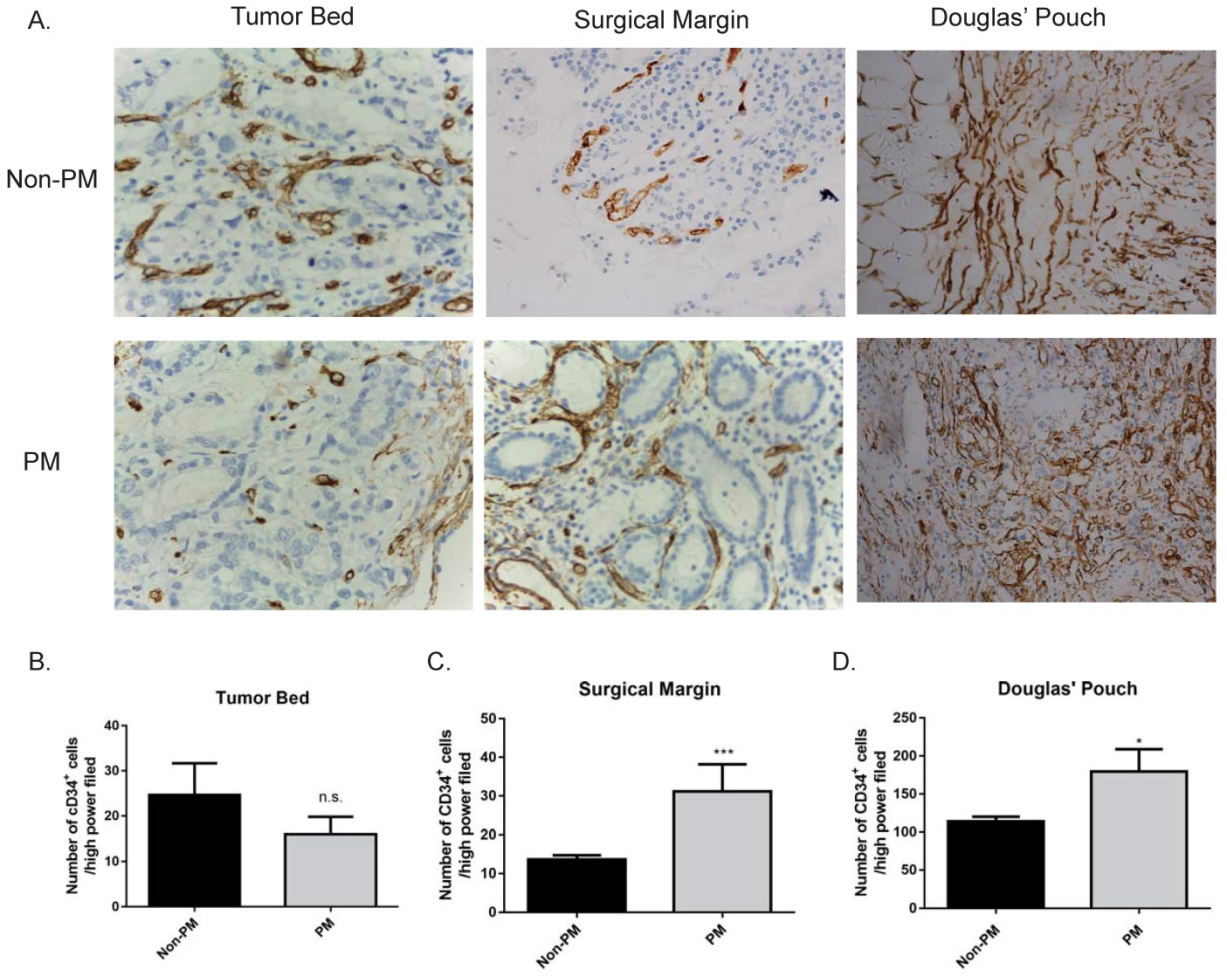
** Expression of CD34 in the primary GC tumor bed, surgical margin and pouch of Douglas.** No difference in the expression of CD34 on the primary tumor bed was observed between the non-metastasis and metastasis group. The expressions of CD34 at the surgical margins and in the Douglas' Pouch in metastasis group were higher than those of non-metastasis group, respectively. A. The expression of CD34 at different sites (400×). B, C, and D, quantification of CD34 expression in different sites. B. tumor bed; C. Surgical margin; D. Douglas' Pouch. PM: peritoneal metastasis. n.s., no significance; *, *p*<0.05; ***, *p*<0.001, by student *t* tests.

During the process of metastasis, angiogenesis plays an important role not only in the primary tumor site (tumor cells invading into the bloodstream), but also the metastatic site (supporting metastatic tumor cells) 28, 29. We stained CD34, a marker for angiogenesis, in the primary tumor bed, surgical margin, and peritoneum by IHC. Expression of CD34 in the primary tumor bed had no difference between peritoneal metastasis patients and non-metastasis patients (data not shown). However, GC patients with peritoneal metastasis had overexpressed CD34 on the surgical margin (no metastasis v.s metastasis, 13.6±1.122 v.s. 31.2±6.989 /HPF, *p*<0.001) and the Pouch of Douglas (no metastasis v.s. metastasis, 113.8±6.256 v.s. 179.4±29.45 /HPF, *p*<0.05) than those of non-metastasis patients (Fig. 4). These data suggested that angiogenesis in the leading edge of the tumor may be involved in the process of tumor cell entering the bloodstream. More importantly, at the peritoneum, angiogenesis plays a supportive role during metastatic tumor cell plantation.

### Macrophages from the peritoneal cavity of GC patients with peritoneal metastasis have an increased capacity to secrete VEGF and EGF

**Figure 5:**
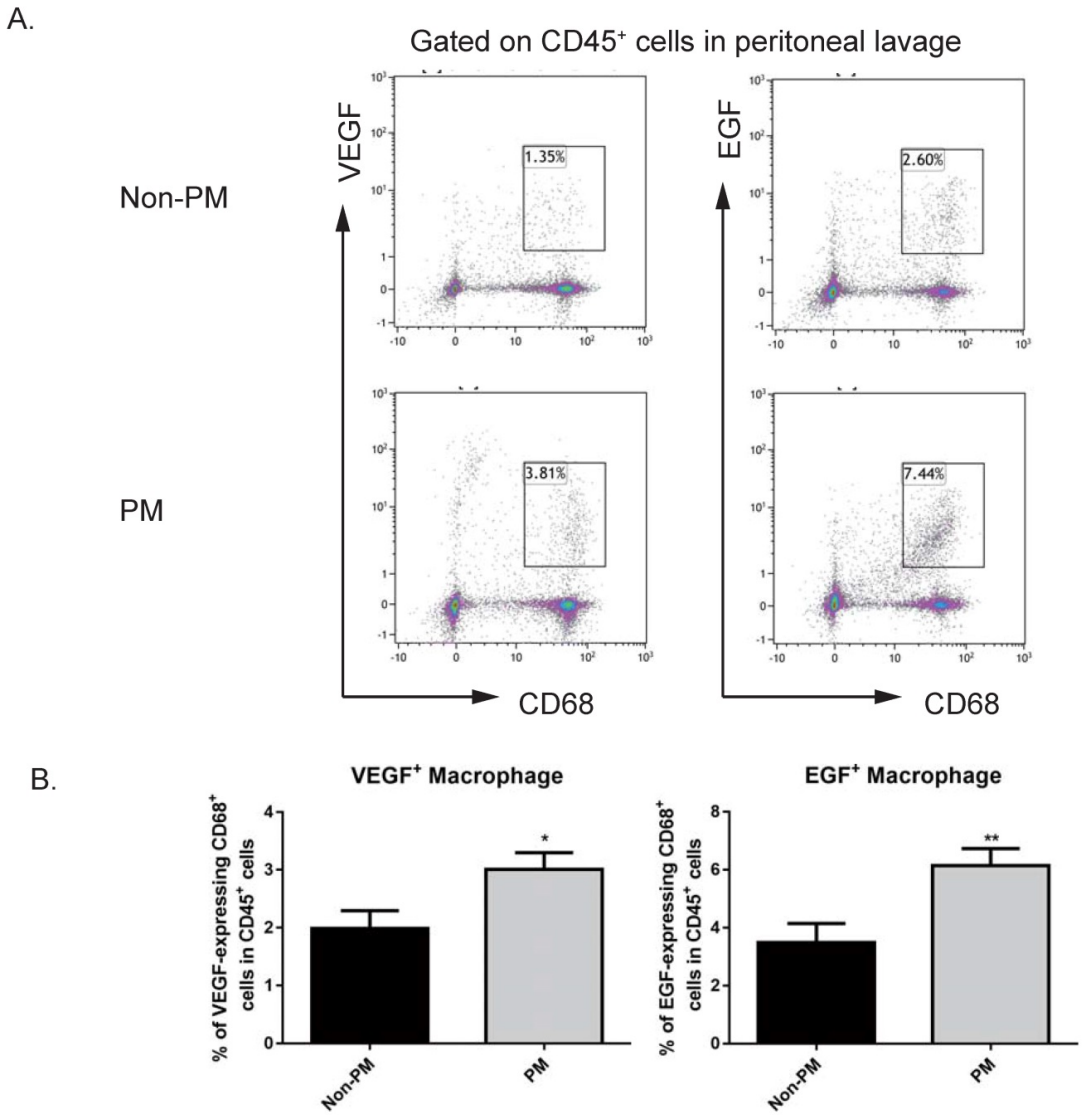
** GC patients with peritoneal metastasis had increased levels of VEGF-expressing and EGF-secreting CD68^+^ macrophages in peritoneal lavage.** Peritoneal washes were collected from GC patients. Macrophages in peritoneal lavage were labelled with CD45 and CD68. EGF- and VEGF-expressing macrophages were intracellularly stained with corresponding antibodies. A. Representative images of flow cytometry analysis. B. GC patients with peritoneal metastasis had more VEGF-secreting macrophages and EGF-expressing macrophages in peritoneal washes compared to patients with no metastasis. n=20. PM: peritoneal metastasis. *, *p*<0.05; **, *p*<0.01, by student *t* tests.

Since VEGF is an imperative factor in angiogenesis 28, 29, and it has been reported that EGF is an active player in TAM-mediated tumor progress 30, we hypothesized that macrophages that were observed to be more abundant on the peritoneum of GC patients with peritoneal metastasis might be a cellular source of VEGF and EGF. In order to test this, we collected macrophages from the first round of peritoneal lavages from GC patients with or without peritoneal metastasis, as published before 26. By utilizing flow cytometry, VEGF-expressing CD68^+^ macrophages and EGF-expressing CD68^+^ macrophages from peritoneal lavages of GC patients with or without peritoneal metastasis were assessed. As shown in Figure 5, GC patients with peritoneal metastasis harbored an increased proportion of VEGF-expressing macrophages in CD45^+^ leukocytes collected from peritoneal lavage, compared to GC patients with peritoneal seeding (no metastasis v.s. metastasis, 1.988±0.306% v.s. 3.008±0.289%, *p*<0.05). Similarly to VEGF-expressing macrophages, GC patients with peritoneal metastasis also had an elevated proportion of EGF-expressing macrophages (no metastasis v.s. metastasis, 3.48±0.666% v.s. 6.14±0.589%, *p*<0.01). These data indicate that macrophages from the peritoneum are a cellular source of EGF and VEGF, which subsequently support tumor progression in the distant metastatic site—the peritoneum.

## Discussion

In this paper, we describe the functions of macrophages in the peritoneum--a common site where metastasis occurs in GC patients: (1) GC patients with peritoneal metastasis had an increased number of macrophages and M2 macrophages in the peritoneum using the Pouch of Douglas as a surrogate; (2) elevated numbers of macrophages in the peritoneum were associated with a poorer prognosis in GC patients; (3) GC patients with peritoneal metastasis had upregulated levels of angiogenesis on the peritoneum; and (4) macrophages isolated from the peritoneal lavage of GC patients with peritoneal metastasis had more EGF- and VEGF-expressing macrophages. These data highlighted the importance of macrophage in the peritoneum in supporting metastatic tumor cells.

Since the relationship between cancer and inflammation has been described by Virchow in the 19^th^ century, an increasing number of studies have been performed for the functions of inflammation in cancer development 31, 32. However, less is known about the inflammation caused by cancer—cancer-related inflammation 13, 15. The cancer is composed of two parts—tumor cells and stromal cells. Tumor cells have gained so much attention and interest from medical doctors and researchers, but recently accumulating evidence demonstrates that cancer-related inflammation supports the development of cancer in various ways. Many components of cancer-related inflammation, including cytokines, chemokines, growth factors and immune cells, play supportive roles to cancer cells. Cancer cells can also recruit leukocytes to the tumor microenvironment in a selective way to choose suitable types of leukocytes for tumor growth. Thus, tumor cells and immune cells built an environment for a mutual interest.

Macrophages in cancer stroma have several mechanisms to promote the growth of tumor cells 33, 34. Macrophages are mainly categorized into two phenotypes--M1 (classically activated macrophages, CAM) and M2 (alternatively activated macrophages, AAM) macrophages 35. M1 macrophages demonstrate a pro-inflammatory phenotype by expressing pro-inflammatory cytokines and presenting antigens information to adaptive immune cells, whereas M2 macrophages show an anti-inflammatory phenotype with the production of anti-inflammatory cytokines. M2 macrophages have an important role during cancer development as a source of growth factors (EGF and VEGF) that proliferate tumor growth and angiogenesis, anti-inflammatory cytokines that control epithelial cells proliferation and wound healing, as well as proteases that promote tumor metastasis 34.

**Figure 6:**
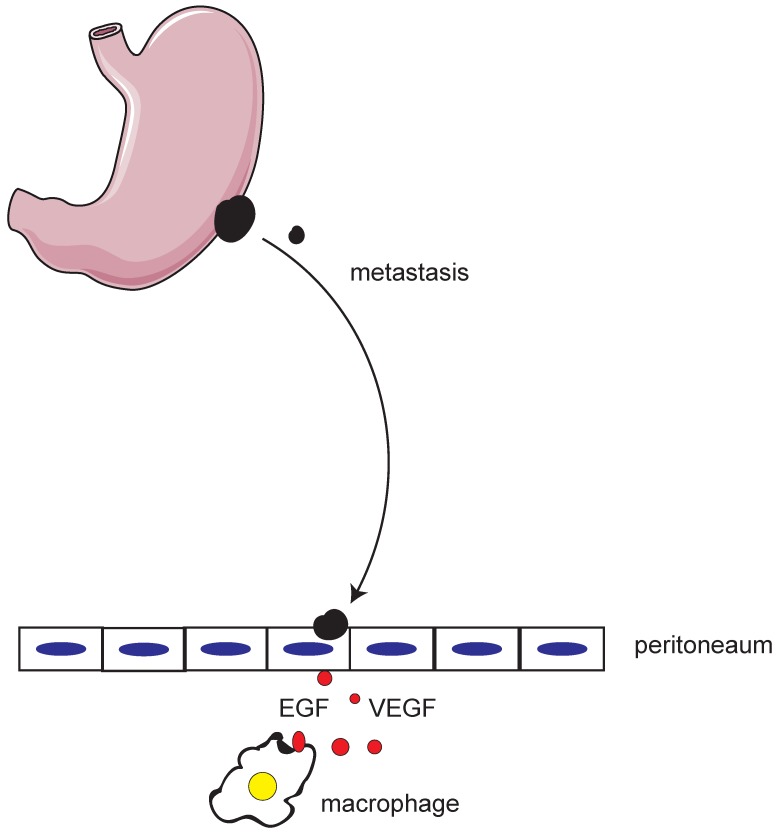
** The roles of peritoneal macrophages in gastric cancer peritoneal metastasis.** When free gastric cancer cells arrive the peritoneum, macrophages support metastatic cancer cells to progress by producing EGF and VEGF.

To our surprise, we did not find any difference in terms of the abundance of macrophages in the primary tumor sites between GC patients with and without peritoneal metastasis. However, we indeed did detect that GC patients with peritoneal metastasis had an increased number of macrophages and M2 TAM on the peritoneum compared to those without peritoneal metastasis. These findings highlight the not-too-old “seed and soil” theory 36, 37. However, beyond the original theory that the vasculature of the distant organs is suitable for seeding of free cancer cells, the present results extend our understanding on the significance of the local immune environment of the distant organ in tumor metastasis. Due to the heterogeneity of tumors, a certain subset of primary cancer cells can leave the tumor site and enter the bloodstream/the peritoneal cavity to become free cancer cells. These free cancer cells “wonder” in the host and “seek” a favorable organ to grow a new metastatic tumor. One factor that free cancer cells seek might be the immune environment. In this study particularly, free cancer cells might find the peritoneum to be a perfect location to grow, where a great number of macrophages reside. Macrophages on the peritoneum produce growth factors, for instance, VEGF and EGF, to support angiogenesis and tumor growth, respectively (Figure 6). Thus, macrophages on the peritoneum build a place of “rich soil” for metastasis. However, it remains unknown whether this characteristic of enriched macrophages in the metastatic location is specific to GC peritoneal metastasis or is a common feature for metastatic cancer. We were not able to describe the abundance of macrophages in other organs, like the liver and lungs, in GC patients. We think it is valuable to characterize the immune environment in different organs in cancer metastasis animal models in the near future.

The abundance of macrophages in the peritoneum can also be used as a marker to predict the prognosis of GC patients. We divided our cohort into two groups based on the number of macrophages per HPF in the peritoneum. Patients bearing a higher number of macrophage (>25/HPF) had a poorer prognosis with the comparison of patients with a fewer number of macrophages in the peritoneum (<25/HPF). To our best knowledge, this is the first study to show the association between the macrophage abundance in the peritoneum and the prognosis in GC patients. It is noteworthy that only a small fraction of GC patients receive a peritoneum biopsy before surgery, which is not a routine test for GC patients. A test using the peritoneal lavage to count macrophages prior to the surgical procedure might be a more feasible strategy. However, a well-designed prospective study is warranted for the prognostic value of macrophage counts in the peritoneal wash. Moreover, in this study, we only recruited T3 GC patients in order to make this study cohort more comparative. One consequence of this cohort is the conclusion of this study is only suitable for T3 GC patients, although we got significant results from this selected cohort. In order to extend our current understanding of peritoneal macrophages in the progression of GC, further clinical observations, as well as animal experiments, are mandated. For this reason, we did not perform a Logistic Regression analysis using the abundance of macrophages, nor the diagnostic value for peritoneal metastasis. Our team is planning a prospective study to assess the prognostic value of macrophages in the peritoneum or peritoneal cavity in GC patients with all stages.

Another question we could not answer in this study is how macrophages are recruited to the metastatic site or which one of more macrophages and peritoneal dissemination is the cause and consequence. It is possible that free cancer cells retain in the peritoneum and then recruit macrophages by secreting chemotactic factors, which in turn favor the growth of metastatic cancer cells. However, it is still possible that resident macrophages in the peritoneum promote metastasis.

In this study, we proved that macrophages in the peritoneum are related to GC peritoneal metastasis. Macrophages in the peritoneum support angiogenesis and tumor growth by producing VEGF and EGF. More importantly, GC patients with more macrophages in the Pouch of Douglas have a poorer prognosis. In conclusion, macrophages in the peritoneum are an active player in GC peritoneal metastasis, which can be used as a therapeutic target in the future based on a deeper understanding of their pathophysiological functions.

## Figures and Tables

**Table 1:** Clinicopathological characteristics of patients

	Peritoneal metastasis group (n=200)	Non-metastasis group (n=200)	*p* Value
Gender—no. (%)			
Male	120 (60%)	120 (60%)	>0.9999
Female	80 (40%)	80 (40%)	
Age—no. (%)			
<65	150 (75%)	110 (55%)	0.323
≥65	50 (25%)	90 (45%)	
Tumor Location—no. (%)			
Middle third	160 (80%)	160 (80%)	>0.9999
Lower third	4 (20%)	40 (20%)	
Maximal length of tumor—no. (%)			
< 5 cm	0 (0)	20 (10%)	0.4872
≥ 5 cm	200 (100%)	180 (90%)	
Macroscopic Types—no. (%)			
3	160 (80%)	160 (80%)	>0.9999
4	40 (20%)	40 (20%)	
Histological Types—no. (%)			
Differentiated	22 (11%)	111 (55.5%)	<0.0001
Undifferentiated	178 (89%)	89 (45.5%)	
Resection —no. (%)			
R 0	0 (0)	151 (75.5%)	<0.0001
R 1	200 (100%)	49 (24.5%)	
Overall Post-surgery Survival (month)			
Range	3-12	36-60	<0.0001
Median	8.7	56.25	

**Table 2:** Details of antibodies used immunohistochemistry staining

	Clone	Manufacture	Titration
CD68	KP1	Abcam, Shanghai, China	1:250
CD163	10D6	Abcam, Shanghai, China	prediluted
CD34	QBEnd-10	DakoCytomation, Carpinteria,CA	1:25
